# Procedural pain and patient-reported side effects with weekly injections of subcutaneous Methotrexate in children with rheumatic disorders

**DOI:** 10.1186/1546-0096-12-54

**Published:** 2014-12-19

**Authors:** Melanie Anne Bechard, Julie Rachelle Lemieux, Johannes Roth, Karen Watanabe Duffy, Ciaran Maire Duffy, Mary Ombac Aglipay, Roman Jurencak

**Affiliations:** University of Toronto Faculty of Medicine, Toronto, Canada; Children’s Hospital of Eastern Ontario Division of Rheumatology, 401 Smyth Road, Ottawa, K1H 8L1 Canada; University of Ottawa, 451 Smyth Road, Ottawa, K1H 8M5 Canada

## Abstract

**Background:**

Despite the widespread use of subcutaneous methotrexate in treating pediatric rheumatic disorders, the amount of pain associated with the injections has not been quantified. Our study aims 1) to quantify the amount of pain associated with subcutaneous injections of methotrexate, 2) to explore predictors of pain, 3) to determine the frequency of patient-reported clinical adverse effects of methotrexate, and 4) identify coping strategies of patients and caregivers.

**Methods:**

Patients aged 4–17 years with rheumatologic diseases who were receiving weekly subcutaneous methotrexate injections for at least 4 weeks were invited to participate in this prospective cohort study. They were trained to use the Faces Pain Scale – Revised (FPS-R) and Faces, Legs, Arms, Cry, Consolability (FLACC) tools to rate pain associated with the injections. All patients underwent focused interviews exploring their experiences with methotrexate injections.

**Results:**

Forty-one patients consented to the study. The mean age was 11.2 years (SD = 3.9 years) and 68% were female. Most patients were diagnosed with JIA (73%). Mean duration of methotrexate therapy was 2.5 years (SD = 2.1 yrs). All but one of the patients used methotrexate 25 mg/ml solution for injection in 1 cc or 3 cc syringe with 30 gauge ½” needle. Median amount of pain was 2/10 on the FPS-R and 1/10 on the FLACC. Higher intensity of pain was significantly associated with presence of side effects (p = 0.004), but not duration of therapy (p = 0.20) or age (p = 0.24). Most participants (61%) experienced at least one adverse effect; nausea (56%) and vomiting (34%) were the most common symptoms reported. Patients and caregivers reported using ice (34%), comfort positions (51%), rewards (49%), reassurance (54%), distraction (51%), and analgesic medications (22%) to cope with the injections.

**Conclusion:**

Subcutaneous injections of methotrexate are associated with a mild amount of pain. Presence of side effects may amplify the amount of perceived pain. Clinicians can apply this knowledge when counseling patients and family members about methotrexate therapy.

## Background

Methotrexate has been an important component of treatment for paediatric rheumatologic conditions for over twenty years [1, 2]. According to the German Pediatric Rheumatology 1998 database, one in four patients diagnosed with chronic rheumatologic diseases received methotrexate treatment [3]. When prescribed to treat these conditions, the recommended dose of methotrexate is 0.3 to 1.0 mg/kg/week (maximal dose 25–30 mg/week)via oral or subcutaneous routes. Weekly methotrexate injections can be associated with increased anxiety in patients with Juvenile Idiopathic Arthritis [JIA; 4], which may be partly attributed to the injection pain and adverse effects.

Procedural pain in paediatric populations is a concern for clinicians due to its association with greater pain and anxiety in subsequent painful events, increased adult pain sensitivity, and long-term avoidance of medical treatment [4, 5]. For example, Weisman et al. [6] have shown that children who do not receive proper pain control during lumbar punctures and bone marrow aspiration report higher amounts of pain for subsequent procedures, even when adequate analgesia has been provided. Additionally, some patients may develop needle phobia and experience disproportionate amounts of fear or vasovagal syncope when presented with needles [7].

Recognition of the negative consequences of inadequately controlled paediatric pain has generated increased interest in pain assessment and management for young patients [8]. A large portion of this research has focused on reducing the pain of common paediatric procedures, such as routine childhood immunizations, venipuncture, intramuscular injections, and dressing changes [9, 10]. To our knowledge, there has not yet been a formal attempt to quantify and reduce the amount of pain associated with subcutaneous methotrexate injections in the paediatric rheumatology population. These patients may be particularly vulnerable to the long-term consequences of uncontrolled procedural pain due to their young ages and relatively long duration of therapy. A recent, large study of children with JIA receiving methotrexate from an average age of 6.1 years, for a median duration of 29 months, found that methotrexate had a detrimental impact on the patients' quality of life [11]. Therefore, there is a need to improve the experience of subcutaneous methotrexate administration. Measuring the amount of pain associated with methotrexate provides a baseline for determining the efficacy of interventions and can aid clinicians in counseling patients and families about to begin therapy.

Given the negative long-term effects of repeated exposure to procedural pain and the vulnerability of the paediatric rheumatology population using subcutaneous methotrexate, our study aimed to address gaps in the existing literature by investigating the following objectives:

1. quantify the amount of pain associated with subcutaneous methotrexate injections,
2. investigate the factors that influence the amount of perceived pain and identify effective pain management strategies used by patients and families, and
3. determine the frequency of clinical adverse effects of subcutaneous methotrexate and effective treatments as reported by the patients and families, and
4. identify coping strategies of patients and caregivers.

## Methods

### Study design

Observational prospective cohort study.

### Study population

All patients at the Children's Hospital of Eastern Ontario Rheumatology Clinic with appointments scheduled between June and August 2013 were screened for their eligibility to participate in this study.

### Inclusion and exclusion criteria

Patients were invited to participate in the study if they were between the ages of 4 and 17 and were currently receiving subcutaneous injections of methotrexate for at least 4 weeks.

### Primary outcome

The primary outcome was the amount of pain associated with subcutaneous injections of methotrexate.

### Secondary outcomes

The secondary outcomes were the 1) predictors of injection-induced pain, 2) patient/family-reported side effects associated with subcutaneous methotrexate, and 3) effectiveness of treatments for these side effects.

### Data collection

1. *Clinical Interview*. A research assistant conducted a standardized focused interview with the patient and family members to ask about their experience with subcutaneous administration of methotrexate. The interview consisted of 18 questions which were developed specifically for this study based on a review of the literature. The interview required approximately 10 minutes to complete.
2. *Chart Review*. Charts were reviewed to gather information pertaining to medical diagnoses and therapy.
3. *Pain Measurements*:
  Participants were taught to how to use the FPS-R to self-report pain, while caregivers were instructed in the use of the FLACC scale to rate their children's pain. One copy of each scale was provided for each of the next two methotrexate injections to increase the accuracy of the assessment. Participants then returned the completed scales via mail or electronic messaging to a single, secure address. An electronic messaging reminder was sent four weeks after the interview if the scales had not already been submitted.
  a/ *Faces Pain Scale* – *Revised* (*FPS*-*R*). This scale consists of six gender-neutral faces depicting “no pain” to “most pain possible”. The child is instructed to point to the face that represents how much pain he/she feels. Ordered faces are scored 0-2-4-6-8-10. This instrument has demonstrated validity and reliability in recording self-reported pain in children between the ages of 4–17 [12, 13].
  b/ *FLACC Behavioral Scale*. This observational scale comprises five items, namely, (F) Face; (L) Legs; (A) Activity; (C) Cry; and (C) Consolability. Each of these five behavioural categories is rated on a scale 0 to 2 to provide an overall pain score ranging from 0 to 10. This tool is valid and reliable for children aged 4–18 [14, 15].

### Statistical analysis

Descriptive statistics were used to analyze the demographics of the patient population and the frequency of the outcomes of interest. The Intraclass Correlation Coefficient was calculated to determine the level of consistency between the FLACC score and Faces Pain Scale. A Bland-Altman plot was also constructed to analyze agreement between instruments. Simple and multiple linear regression were conducted to determine the association between both the FLACC score and Faces Pain Scale and age, presence of side effects, and duration of treatment. All analyses were conducted using SPSS v. 21 and R v. 3.0.2.

### Ethics

This study was approved by the Children's Hospital of Eastern Ontario Research Ethics Board (REB Protocol No 13/98X).

## Results

### Patient demographics

Of the 42 patients who met the inclusion criteria for this study, 41 consented to participate (participation rate 97.6%). The mean age was 11.2 years (SD = 3.9 yrs) and 68.3% of participants were female. Most of these patients were diagnosed with JIA (73.2%; Table 1). Mean duration of therapy with subcutaneous injections of methotrexate was 2.5 years (SD = 2.1 years; Table 1). Throughout the clinical interviews, the primary respondent was most commonly the patient's mother (18/41, 43.9%), followed by the patient herself/himself (13/41, 31.7%). Fathers of the patients were the primary respondents in the remaining interviews (10/41, 24.4%). The majority of participants (82.9%) had no active joints at the time of the clinical interview. Use of subcutaneous methotrexate was discontinued for 4 of the participants shortly after the clinical interview as per the recommendation of their rheumatologist. Two of these patients were advised to discontinue subcutaneous methotrexate because of sustained remission. The other two participants were unable to tolerate the subcutaneous therapy and were therefore switched to oral methotrexate. These children identified their dislike of the injections as their main reason for refusing the subcutaneous therapy. Therefore, 37 patients were trained on the use of the pain scales to rate the amount of pain with future injections. Twenty-nine of the 37 participants returned the completed pain scales (response rate 78.4%).The majority of patients (92.7%) reported excellent adherence to the treatment regimen, missing less than one dose per month.

**Table 1:** **Demographic information of study participants**

Mean age	11.2 years (SD = 3.9 years)		
Mean duration of Methotrexate therapy	2.5 years (SD = 2.1 years)		
Sex	Female:	28/41	68%
Rheumatologic diagnosis*	JIA:	30/41	73%
	Uveitis:	14/41	34%
	Scleroderma:	10/41	24%
	Dermato/Polymyositis:	2/41	5%
	SLE	1/41	2%
	Other	4/41	10%
Number of active joints at time of interview	0 Active Joints:	34/41	82%
	1 Active Joint:	5/41	12%
	2 Active Joints:	1/41	2%
	3 Active Joints:	1/41	2%
Non-Methotrexate rheumatologic medications	None:	15/41	37%
	Biologics:	15/41	37%
	Prednisone:	11/41	27%
	NSAID:	5/41	12%
	Methylprednisolone:	2/41	5%
	Other (i.e. topical):	8/41	20%
Folic acid	1 mg daily or 5 mg weekly	29/41	71%
	None	12/41	29%

* Some participants had both JIA and uveitis.

### Subcutaneous Methotrexate injection techniques and setting

All but one of the patients used methotrexate 25 mg/ml solution for injection in 1 cc or 3 cc syringes with 30 gauge ½” needles. One patient used Metoject® 20 mg/2 ml prefilled syringes. The majority of participants received methotrexate injections in the evening (30/41, 73.2%). Weekend days were the most popular for injection administration (31/41, 75.6%). In most families, the child's mother was responsible for administering the injection (24/41, 58.5%), followed by the father (10/41, 24.4%). Only 12.2% of patients regularly injected the medication themselves. The upper arm was the preferred site of injection in 70.7% of patients. Approximately 19.5% of caregivers reported preparing their children for the injection by "counting to three" in advance; the remaining participants did not receive warning before needle insertion.

### Amount of pain associated with subcutaneous Methotrexate

**Figure 1:**
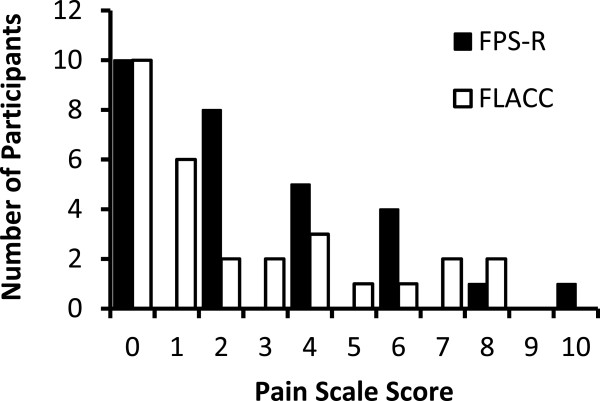
**Amount of self-reported or observational pain as measured on the FPS-R and FLACC.**

The set of pain scores from the first injection was used in statistical analyses. Of the 29 patients who completed the pain scales, the median amount of self-reported pain on the FPS-R was 2/10; 18 (62.1%) patients reported nil to mild pain (FPS-R score 0–2), and 9 (31.0%) reported moderate pain (FPS-R score 4–6). Two participants reported severe pain (FPS-R 8–10; Figure 1). The patient who reported the highest amount of pain was the only one using Metoject® 20 mg/2 ml prefilled syringes and was excluded from further analyses.

Similarly, analysis of observational pain scores from the FLACC resulted in a median score of 1/10. The majority of caregivers (20/29; 68.9%) rated the pain 3/10 or less (Figure 1). Pain scores from the FLACC and FPS-R showed good overall agreement (ICC = 0.87, 95% CI: 0.75-0.94).

### Predictors and alleviators of pain

**Figure 2:**
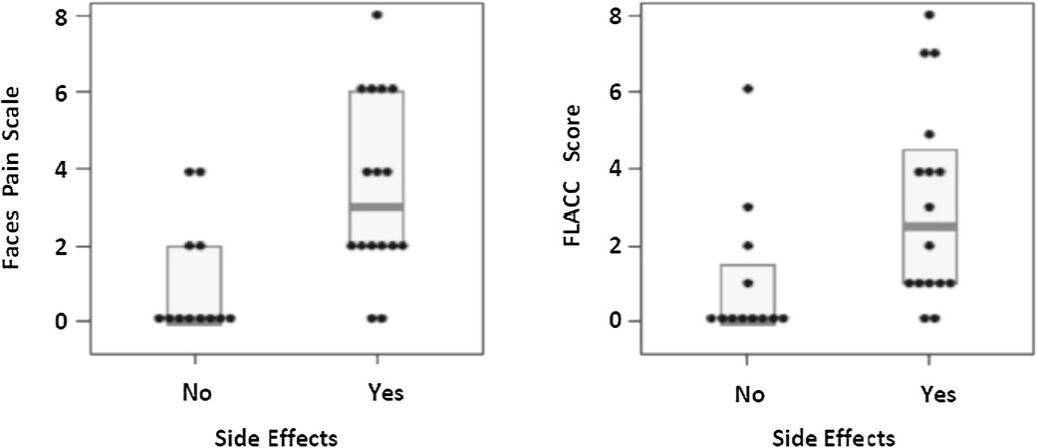
**Gastrointestinal side effects and methotrexate injection pain.** The presence of nausea or vomiting from methotrexate is associated with higher injection pain scores on both the FPS-R and FLACC.

Associations between the amount of self-reported pain on the FPS-R and presence of side effects, duration of therapy, and patient age were tested. In univariate testing, higher intensity of pain was associated with presence of side effects (p = 0.004; Figure 2), but not duration of therapy (p = 0.20) or age (p = 0.24). Results were largely unchanged in multiple linear regression adjusting for all three variables. The presence of side effects was independently associated with a 2.6 unit higher FPS-R (95% CI: 1.0-4.1; p < 0.01). Neither age (0.1 units lower FPS-R per year, 95% CI: 0.4 units lower to 0.1 higher; p = 0.24) nor duration of therapy (0.02 units higher FPS per month, 95% CI: −0.004- 0.05; p = 0.10) were found to be independently associated with FPS-R.

Similar results were found when presence of side effects, duration of therapy, and patient age were tested for association with the amount of pain reported using the FLACC. Univariate analyses demonstrated that higher intensity of pain was associated with the presence of side effects (p = 0.03), but not with duration of therapy (p = 0.45) or age (p = 0.33). Multiple linear regression adjusting for all three variables demonstrated that the presence of side effects was independently associated with a 2.1 unit higher FLACC (95% CI: 0.2-4.0, p = 0.03). Age (−0.10 units lower per year, 95% CI: −0.36-0.16; p = 0.45) and duration of therapy (0.01 units higher per month, 95% CI: −0.02-0.05; p = 0.36) were not significantly associated with FLACC scores.

Participants were asked if they made past or current attempts to reduce the pain associated with the injections through any combination of various pharmacological and psychological techniques. The majority of participants (80.5%) tried at least one method. The frequency of use and reported efficacies of these methods are described in Table 2.

**Table 2:** **Techniques to alleviate pain associated with subcutaneous injections of methotrexate**

Method	Examples	Participants who tried method	Participants who found method effective
Ice	Before and/or after injection	14 (34.1%)	10 (71.4%)
Comfort Positions	Hugging, holding hands	21 (51.2%)	14 (66.7%)
Rewards	Food treats, toys, fun activities	20 (48.8%)	13 (65.0%)
Reassurance	“It will be fast”, “Don’t worry”	22 (53.6%)	11 (50.0%)
Distraction	Music, TV, video games	21 (51.2%)	10 (47.6%)
Medicinal	Advil, Tylenol, EMLA, anesthetic spray	9 (22.0%)	4 (44.4%)

Participants were asked to describe changes in pain intensity of methotrexate injections over the course of therapy. While 7/41 patients (17.1%) reported that the pain has worsened over time, 12/41 patients (29.3%) reported decreased intensity. An additional 19/41 (46.3%) of patients reported no change in pain intensity throughout the course of therapy, and 3/41 (7.3%) of participants were unable to identify any consistent trend.

### Subjective side effects

**Figure 3:**
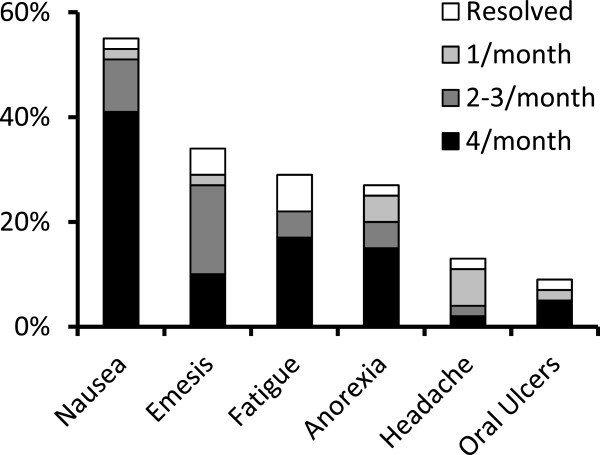
**Self-reported frequency of clinical adverse effects associated with weekly subcutaneous injections of methotrexate.**

Most participants (25/41, 61.0%) experienced at least one clinical adverse effect (Figure 3). The two most commonly reported side effects were nausea and vomiting (56.1% and 34.1% of patients, respectively). Less frequently reported side effects included fatigue (29.2%), anorexia (26.8%), headache (14.6%), and recurrent oral ulcers (9.8%). Of patients who experienced nausea, 52.2% used dimenhydrinate while 26.1% used ondansetron to manage their symptoms. Similarly, many patients who experienced vomiting tried to alleviate their symptoms with dimenhydrinate (57.1%) or ondansetron (28.5%). While dimenhydrinate effectively treated nausea in only 41.7% of patients, most patients reported good efficacy of ondansetron (83.3%). A similar effect was observed for treatment of vomiting (efficacy of dimenhydrinate 12.5% versus ondansetron 75.0%). None of the patients used ginger to alleviate nausea/vomiting.

The timing of onset and the duration of clinical adverse effects were also recorded (Table 3). Anticipatory nausea was experienced by 56.5% of all participants who reported this symptom; the remainder felt nauseous after the injections. Similarly, 42.9% of all reported vomiting was anticipatory. Most reported subjective adverse effects were relatively short-lived. Except for oral ulcers, clinical side effects resolved within 24 hours in 92.7% of patients.

**Table 3:** **Adverse effects related to subcutaneous injections of methotrexate**

Adverse effect	Prevalence	Onset in relation to injection	Number (%)		Duration	Number (%)	
Nausea	23 (56%)	Before	13	(56%)	0 - 5.9 hours	9	(39%)
		During/After (< 1 hour)	6	(26%)	6 - 11.9 hours	4	(17%)
		After (1–6 hours)	2	(9%)	12 - 23.9 hours	9	(39%)
		After (> 6 hours)	5	(22%)	≥ 24 hours	1	(4%)
Vomiting	14 (34%)	Before	6	(43%)	0 - 5.9 hours	12	(86%)
		During/After (< 1 hour)	4	(29%)	6 - 11.9 hours	0	(0%)
		After (1–6 hours)	3	(21%)	12 - 23.9 hours	1	(7%)
		After (> 6 hours)	3	(21%)	≥ 24 hours	1	(7%)
Headache	6 (15%)	< 12 hours	3	(50%)	< 12 hours	5	(83%)
		12 - 24 hours	2	(33%)	12 - 24 hours	1	(17%)
		> 24 hours	1	(17%)	> 24 hours	0	(0%)
Fatigue	12 (29%)				< 12 hours	1	(8%)
					12 - 24 hours	9	(75%)
					> 24 hours	2	(17%)
Anorexia	11 (31%)				< 12 hours	4	(36%)
					12 - 24 hours	6	(54%)
					> 24 hours	1	(9%)

Of 19 patients who had used oral methotrexate in the past, only 26.3% felt that the oral form was better tolerated. The remaining patients either favoured the subcutaneous route (6/19, 31.6%), had no preference (7/19, 36.8%), or were undecided (1/19, 5.3%).

## Discussion

### Subcutaneous injections of methotrexate are associated with a small amount of pain

The participants within our study reported mild pain associated with the weekly subcutaneous methotrexate injections (2/10 on the FPS-R). Interestingly, approximately a third of patients and a third of caregivers reported no injection pain (FPS-R or FLACC score of 0). Compared to other studies that have used the FPS-R to measure pain associated with analogous procedures, our participants reported a relatively low amount of pain. For example, Berberich and Landman [16] measured pain associated with intramuscular and subcutaneous immunizations in children aged 4 to 6 using the FPS-R and FLACC. Without intervention, the pain was rated as 8.00 and 7.00 using the FPS-R and FLACC, respectively. Studies investigating the amount of pain associated with venipuncture in children typically report a median FPS-R score of 4.00 to 7.00 if no prophylactic intervention is applied [17–19]. While differences between study populations may partially account for this higher level of pain, our findings suggest subcutaneous methotrexate injections produce less pain than venipuncture. Another study found that children aged 5 to 12 reported an average pain score of 2.63 on the FPS-R following ear piercing, which is similar to the reported amount of methotrexate injection-associated pain [20]. These comparisons may be useful when counseling parents and children about subcutaneous methotrexate therapy.

Though the median amount of pain reported in our study is relatively low, it is important to note that some children experienced moderate to severe pain with the injections. For example, two of the participants recruited for this study refused to regularly receive the injections due to the associated discomfort. Within our study, 92.7% of participants reported missing less than one dose of methotrexate per month. The rate of reported paediatric adherence to methotrexate therapy (either oral or subcutaneous) within the existing literature is difficult to assess due to various operational definitions for adherence. However, adherence seems to vary from 82% to 95.9% between studies [11, 21]. The real adherence rate in our population may have been much lower than what we have captured through self-report data. It is important for clinicians to understand the challenges some patients face and to be prepared to discuss techniques for pain control in order to optimize adherence and treatment efficacy.

### Clinicians should become familiar with the various available methods of pain control

Families who participated in this study reported using a variety of techniques to alleviate the pain associated with injections. Interestingly, two thirds of our patients who tried ice to cool the skin prior to or immediately after injection found this intervention effective despite a systematic review of physical techniques to reduce injection pain that found inconsistent evidence to support the benefit from ice application [22] and subsequent clinical guidelines for administration of vaccines that found insufficient evidence to recommend the use of ice to reduce injection pain [23]. Further studies are needed on the application of ice for methotrexate injection pain.

Approximately half of our participants reported using comfort positions, such as hugging or hand-holding, to reduce to the injection pain. Two thirds of these patients reported experiencing some degree of pain relief. This is in keeping with prior research reports showing benefits of hugging children during painful procedures [24, 25]. In addition, studies with infants suggest skin-to-skin contact and sitting upright as opposed to supine decrease injection-associated pain [22].

Another common technique our families used to reduce pain involved providing rewards, either in the form of food treats (including sweets), toys, or enjoyable activities. Most of our patients reported experiencing pain relief due to the promise of reward. While there is no comparative study on the effects of promised future rewards on pain, a recent Cochrane review of randomized control studies investigating the use of oral glucose or sucrose for pain relief during needle-related procedures found these techniques did not reduce pain scores in school-aged children [26]. This discrepancy may relate to the self-report nature of our study. For example, the respondents might have said the rewards were helpful because they enjoyed receiving them, even if they did little to relieve pain during the injection itself.

Systematic reviews have reported that distraction is an effective coping method used to alleviate paediatric needle-related pain [27–29]. As expected, about half of our patients reported experiencing pain relief with distraction. There are many different types of distraction; this category may be classified according to who is leading the distraction (parent, child, or health care provider) or the object used to provide distraction itself (bubbles, toys, passive electronics such as television, active electronics such as video games). The efficacy of distraction for pain relief may vary amongst these domains. For example, there is some evidence that child-led or nurse-led distraction decreases pain on more metrics than parent-led distraction [27]. Furthermore, prior studies suggest the efficacy of distraction techniques for pain control depend upon the particular method used and the temperament of the child [29–31]. Our findings are consistent with existing literature suggesting distraction may be an effective method of pain relief.

### Controlling methotrexate induced nausea and vomiting may alleviate injection pain

We found that experiencing nausea or vomiting in association with the methotrexate injections was associated with higher pain scores on the FPS-R and FLACC. These gastrointestinal symptoms are the most common side effects associated with methotrexate therapy and have been linked to a reduced health-related quality of life [11]. It is possible that the distress caused by the anticipation of these side effects heightens anxiety and pain perception.

Given this association, adequate control of these symptoms is desirable for alleviating injection-associated pain and its long-term consequences. Results of our study suggest that dimenhydrinate has only a very limited role in treatment of methotrexate associated nausea and vomiting, and perhaps ondansetron should be the treatment of choice in this situation. There is some evidence that psychological intervention may be beneficial to managing anticipatory nausea and vomiting when methotrexate is prescribed for JIA [32].

### Study limitations

Due to the observational nature and relatively small size of our study, we are unable to strongly recommend for or against particular methods of pain relief. However, each of the techniques described above provided relief for at least some participants. The low cost and low risk of these interventions justifies their trial. The small size of our study also prevented stratification of participants by age, rheumatologic condition, and anxiety. Further research is needed to determine whether the results generalize to different populations within these categories.

## Conclusions

This study provides detailed information on the amount of patient-perceived pain with subcutaneous injections of methotrexate and potential pain aggravating factors. In addition, the paper explores patients' and caregivers' perception of methotrexate-associated side effects and their coping strategies. We hope the results will help clinicians counsel patients and families starting weekly methotrexate injections. The possible association between the presence of gastrointestinal side effects and higher reported amounts of pain warrants further study. Careful management of these adverse effects may help to avoid the negative long-term consequences of inadequately managed procedural pain.

## Consent

We obtained a consent from patients and guardians to participate in the study. We did not obtain a consent for publication of this report as it was not required by our REB.

## Abbreviations

JIA: Juvenile idiopathic arthritis
FPS-R: Faces pain scale - revised
FLACC: Face, legs, arms, cry, consolability
REB: Research ethics board
SD: Standard deviation
SLE: Systemic lupus erythematosus
NSAID: Non-steroidal anti-inflammatory
EMLA: Eutectic mixture of local anesthetics.
